# Successful Management of Life-Threatening Hypernatremia (Na⁺ 186 mmol/L): A Case Report

**DOI:** 10.7759/cureus.93176

**Published:** 2025-09-25

**Authors:** Bedih Balkan, Ebru Kaya, Hatice D Özcanoğlu, Ali Osman Balkan, Gülseren Yılmaz

**Affiliations:** 1 Adult Intensive Care, Istanbul Kanuni Sultan Suleyman Training and Research Hospital, Istanbul, TUR; 2 Intensive Care Unit, Istanbul Kanuni Sultan Suleyman Training and Research Hospital, Istanbul, TUR; 3 Department of Anesthesiology and Reanimation, Istanbul Saglık Bilimleri University Basaksehir Cam and Sakura Hospital, Istanbul, Turkey, Istanbul, TUR; 4 Department of Internal Medicine, University of Pittsburgh Medical Center (UPMC) McKeesport, Pittsburgh, USA; 5 Department of Anesthesiology and Reanimation, Kanuni Sultan Suleyman Research Institue, Istanbul, TUR

**Keywords:** cerebral edema prevention, electrolyte imbalance, fluid therapy, intensive care, severe hypernatremia

## Abstract

Hypernatremia, defined as a serum sodium level greater than 145 mmol/L, is a frequent electrolyte disorder, affecting approximately 1%-3% of hospitalized patients and associated with high morbidity and mortality, with rates exceeding 40% in severe cases. Severe cases, with levels above 180 mmol/L, are generally considered fatal. We describe the case of a 21-year-old bedridden male with a congenital intellectual disability and epilepsy who presented with a three-day history of vomiting, diarrhea, decreased intake, and lethargy. On admission, he was hypotensive, tachycardic, dehydrated, and somnolent, with a serum sodium level of 186 mmol/L. Initial management focused on restoring his intravascular volume with isotonic saline, followed by gradual correction using hypotonic fluids. This was done while ensuring the rate of sodium reduction did not exceed 10-12 mmol/L per day. Electrolytes were monitored closely, and seizure precautions were implemented throughout his treatment. The patient’s neurological status improved progressively with the stepwise sodium correction, and he ultimately recovered fully without complications. This case highlights that even extreme hypernatremia can be reversed with careful hemodynamic stabilization, meticulous monitoring of electrolytes, and optimized fluid management, which can lead to favorable outcomes in vulnerable patients.

## Introduction

Hypernatremia, defined as a serum sodium level greater than 145 mmol/L, is a frequent electrolyte disorder, affecting approximately 1%-3% of hospitalized patients and associated with high morbidity and mortality, with rates exceeding 40% in severe cases, particularly in vulnerable patients with limited access to fluids. The leading causes are a relative water deficit and excessive solute accumulation. The most common cause is a loss of total body water that outpaces the loss of solutes. Hypernatremia is often linked to hypovolemia and can occur when water loss surpasses sodium loss or free water loss, leading to a combined depletion of water and solutes. These combined losses can result from non-renal conditions such as gastroenteritis, vomiting, prolonged nasogastric drainage, burns, and excessive sweating. Excessive sweating can be a consequence of exercise, fever, or exposure to high temperatures. While severe cases with serum sodium levels exceeding 180 mmol/L are often considered fatal, emerging evidence suggests that careful management can lead to survival. The underlying mechanism involves a water deficit relative to sodium, which results in cerebral dehydration and severe hemodynamic instability. The primary treatment approach involves slow correction to prevent rapid fluid shifts that could cause dangerous cerebral edema [1-4]. This review outlines the principles for managing such critical cases, emphasizing that a fatal outcome is not inevitable even with extremely high sodium levels [5]. This case report of a patient with extreme hypernatremia (Na+ 186 mmol/L) underscores the importance of a structured approach, which includes initial stabilization of circulation, gradual sodium reduction, and close monitoring of this potentially life-threatening electrolyte imbalance. Prompt but cautious treatment is crucial to prevent brain swelling and neurological damage.

## Case presentation

We present the case of a 21-year-old bedridden man with a congenital intellectual disability and epilepsy who developed severe hypernatremia (serum sodium 186 mmol/L) secondary to recurrent vomiting and diarrhea. His past medical history included congenital mental retardation and epilepsy, for which he was receiving valproic acid. He was admitted to the emergency department after three days of persistent vomiting, watery diarrhea, markedly reduced oral intake, and progressive lethargy. The patient was nonverbal and fully dependent on caregivers for his daily needs.

On arrival, his blood pressure was 85/55 mmHg, heart rate 132 beats per minute, body temperature 37.6 °C, and respiratory rate 28 breaths per minute with shallow effort. Oxygen saturation was 89% on room air, which improved to 94% with supplemental oxygen. The physical exam revealed dry mucous membranes, decreased skin turgor, and a delayed capillary refill of more than three seconds. A neurological assessment showed profound somnolence with a Glasgow Coma Scale (GCS) score of 7 (E2, V1, M4); the patient withdrew only to painful stimuli and did not follow commands. Due to the combination of severe encephalopathy, inability to protect his airway, and worsening hypoxemia, he was intubated and mechanically ventilated.

Initial management focused on restoring intravascular volume and gradually correcting the serum sodium to prevent cerebral edema. Hemodynamic stabilization was attempted with repeated boluses of isotonic saline (0.9% NaCl), followed by a maintenance infusion. After stabilization, the free water deficit was calculated, and fluid therapy was transitioned to hypotonic solutions (0.45% NaCl combined with 5% dextrose) to allow for a slow sodium reduction (Table 1). The correction target was strictly limited to no more than 10-12 mmol/L per 24 hours to mitigate the risk of cerebral edema. Serial monitoring included serum sodium measurements every four hours, strict input-output tracking, and close neurological assessment while the patient remained under mechanical ventilation. After the fifth day of the ICU stay, the patient's serum sodium level was successfully corrected to 140 mmol/L. Concurrently, his respiratory parameters stabilized, and his level of consciousness and responsiveness improved significantly. He was extubated on the fifth day and was transferred to the neurology ward for continued care on the seventh day.

**Table 1:** Stepwise laboratory changes during hypernatremia correction with normal reference ranges. Na, sodium; K, potassium; Cl, chloride; BUN, blood urea nitrogen; GCS, Glasgow Coma Scale

Parameter	Reference range	0 hours	24 hours	48 hours	72 hours
Na⁺ (mmol/L)	135-145	186	174	162	148
K⁺ (mmol/L)	3.5-5.0	3.4	3.6	3.8	4.0
Cl⁻ (mmol/L)	98-106	143	138	134	102
Osmolality (mOsm/kg)	275-295	410	388	360	310
BUN (mg/dL)	7-20	140	112	82	46
Creatinine (mg/dL)	0.6-1.3	1.8	1.5	1.2	0.8
Neurological status (GCS)	Normal = 15	Obtunded (GCS 9)	Obeys simple commands	Conversant, oriented	Baseline cognition

## Discussion

Severe hypernatremia management in a young adult with intellectual disability

Severe hypernatremia, defined as serum sodium levels above 160 mmol/L, is associated with high morbidity and mortality, particularly in elderly, critically ill, or neurologically impaired patients. This condition reflects a water deficit in relation to sodium and is often a result of gastrointestinal losses, renal concentrating defects, or limited access to free water [2,6]. Rapid correction poses significant risks, including cerebral edema, seizures, and even death, especially when chronic or of uncertain duration [7].

We present a case of a young adult with an intellectual disability who developed severe hypernatremia (Na⁺ 186 mmol/L) due to repeated vomiting and diarrhea. Individuals with neurological impairments and dependence on caregivers are particularly vulnerable due to impaired thirst perception and limited ability to access fluids [8,9].

Management strategy

Management of hypernatremia involves three critical steps:

Hemodynamic stabilization: Initial treatment consists of administering isotonic fluids to restore hemodynamic stability and prevent hypovolemic shock.

Calculation of free water deficit: Assessing the free water deficit is essential for fluid replacement strategies.

Controlled correction of serum sodium: Correction should be gradual, typically not exceeding 0.5 mmol/L per hour or 10-12 mmol/L per 24 hours when chronicity is uncertain.

Close monitoring of serum sodium levels, fluid balance, and neurological status is essential during therapy. In cases of acute hypernatremia (onset less than 48 hours), more rapid correction is acceptable; however, when the duration is unclear, a cautious approach is recommended [10,11]. Our patient's management was conducted in accordance with established guidelines that prioritize hemodynamic stabilization followed by gradual correction with isotonic and hypotonic fluids. Strict adherence to the recommended sodium correction rate and intensive monitoring every four hours played a key role in preventing neurological complications and ensuring a successful recovery.

Comparative outcomes

Global reports support the efficacy of cautious approaches to hypernatremia. For instance, research by Alharfi et al. demonstrated survival in pediatric patients with sodium levels exceeding 190 mmol/L due to controlled, slow correction [12]. A case report by Carlberg et al. highlighted the survival of an adult with an initial sodium level of 196 mmol/L, underscoring the importance of avoiding rapid fluid shifts [13]. Our case contributes to evidence challenging the perception of inevitable fatality at levels above 180 mmol/L and affirms the effectiveness of standardized guideline approaches.

Preventive strategies and future directions

This case emphasizes the importance of preventive strategies. Close monitoring of fluid intake, output, and neurological status is crucial during illnesses such as vomiting or diarrhea. For the future, it is recommended that caregivers receive increased education on recognizing the early signs of dehydration and ensuring adequate fluid intake. Additionally, future research could focus on developing refined protocols for distinguishing between acute and chronic hypernatremia in comatose patients, possibly through novel biomarkers or imaging techniques, such as diffusion-weighted MRI, to assess neuronal stress [14].

## Conclusions

In summary, this case demonstrates that severe hypernatremia (serum sodium >180 mmol/L) can be safely treated using structured, evidence-based methodologies. It highlights the critical vulnerability of individuals, particularly those with neurological impairments who rely on caregivers for hydration, to the risk of dehydration during common illnesses. Therefore, educating caregivers of high-risk patients to recognize early signs of dehydration and to ensure adequate fluid intake is essential. Furthermore, there is a need for future research to develop more precise protocols for differentiating acute from chronic hypernatremia in comatose patients, potentially utilizing innovative biomarkers or imaging techniques such as diffusion-weighted MRI. These approaches will enhance patient safety and optimize treatment processes.
